# Phosphodiesterase-5 Inhibitors: Action on the Signaling Pathways of Neuroinflammation, Neurodegeneration, and Cognition

**DOI:** 10.1155/2015/940207

**Published:** 2015-12-03

**Authors:** Christina Alves Peixoto, Ana Karolina Santana Nunes, Ana Garcia-Osta

**Affiliations:** ^1^Laboratório de Ultraestrutura, Centro de Pesquisa Aggeu Magalhães (FIOCRUZ), 50.740-465 Recife, PE, Brazil; ^2^Universidade Federal de Pernambuco, 50.670-901 Recife, PE, Brazil; ^3^Neurobiology of Alzheimer's disease, Neurosciences Division, Center for Applied Medical Research (CIMA), University of Navarra, Pamplona, Spain

## Abstract

Phosphodiesterase type 5 inhibitors (PDE5-Is) have recently emerged as a potential therapeutic strategy for neuroinflammatory, neurodegenerative, and memory loss diseases. Mechanistically, PDE5-Is produce an anti-inflammatory and neuroprotection effect by increasing expression of nitric oxide synthases and accumulation of cGMP and activating protein kinase G (PKG), the signaling pathway of which is thought to play an important role in the development of several neurodiseases, such as Alzheimer's disease (AD), Parkinson's disease (PD), and multiple sclerosis (MS). The aim of this paper was to review present knowledge of the signaling pathways that underlie the use of PDE5-Is in neuroinflammation, neurogenesis, learning, and memory.

## 1. Introduction

The PDE superfamily consists of 11 subtypes (PDE1–PDE11), the classification of which is based largely on their sequence homology. PDE1, PDE2, PDE3, PDE10, and PDE11 hydrolyse cGMP and cAMP, PDE4, PDE7, and PDE 8 preferentially cleave cAMP, while PDE5, PDE6, and PDE9 cleave cGMP. PDE5 is highly expressed in the cerebellar Purkinje cells [1] and is also expressed in other brain areas such as the hippocampus, caudate, substantia nigra, and cerebellum [2, 3]. There are indications that PDEs can affect neuronal cell survival and when functioning incorrectly they may play a part in neurodegenerative diseases, such as Alzheimer's disease, major depression disorder, multiple sclerosis, Huntington's disease (HD), and Parkinson's disease [4].

Inhibition of specific PDE5s (PDE5-Is) and accumulation of cGMP may inhibit neuroinflammation and improve synaptic plasticity and memory [5, 6]. Among the compounds synthesized and screened against PDE5, the most utilized drugs in neuroinflammation/neurodegeneration assay are the cGMP based sildenafil (VIAGRA, Pfizer) and vardenafil (Levitra, Bayer HealthCare Pharmaceuticals) and the *β*-carboline derived tadalafil (Cialis, Eli Lilly Laboratories). The possible use of these drugs in the central nervous system (CNS) is related to their ability to cross the blood-brain barrier (BBB). Sildenafil has been described as clearly crossing the BBB (FDA 1998) and there is also evidence of the ability of vardenafil to do the same [7, 8]. According to Akkerman et al. [9] the neuropharmacokinetic (plasma and brain) profile of vardenafil after oral administration is detected within 4 min after dose [9]. Until recently it was considered that tadalafil was unable to cross the blood-brain barrier (BBB) [10], but the most recent results have shown that tadalafil reaches the brain in sufficient concentrations to potently inhibit PDE5 [11].

In recent years there has been tremendous interest in the potential therapeutical use of PDE5-Is in the treatment of several diseases. There is mounting evidence from clinical and experimental trials that indicates that NO-cGMP-PKG is the central mechanism of a network of signaling pathways that interconnects neuroinflammation, neurodegeneration, and cognitive disorders, resulting in increased pharmaceutical interest in PDE5-Is as promising therapeutic targets for neurodiseases.

## 2. Neuroinflammation/Neurodegeneration

### 2.1. Blood-Brain Barrier

Neuroinflammation is an inflammation of the nervous tissue that can be initiated in response to a variety of stimuli, including infection, head injury, toxic metabolites, or autoimmunity, and has a pivotal role involved in the progression of neurodegenerative disorders. Until recently the CNS was considered immunologically privileged, as many antibodies and peripheral immune cells are blocked by the blood-brain barrier (BBB), a highly specialized brain endothelial structure composed of pericytes, astrocytes, and microglia. The BBB maintains the chemical composition of the neuronal environment, which is required for the proper functioning of the neuronal circuits, synaptic transmission, synaptic remodeling, angiogenesis, and neurogenesis [12]. The immune system influences the functioning of the BBB, which, in turn, affects the functioning of the CNS under both physiological and pathological conditions. In some cases, the BBB separates the CNS from the immune system; in others it acts as a mediator in neuroimmune interaction, and still in others it may act as an immune system attack target [13]. Under physiological conditions, immune cells cross the BBB at a very low rate through specific interactions [14, 15]. In neurodegenerative disorders, however, the BBB is impaired. The inflammatory process is initiated when the glial cells are activated, with a consequent change in the transendothelial transport of monocytes and T lymphocytes, the secretion of cytokines, and finally neuronal damage and death. Inflammatory cytokines such as TNF-*α* and IL1-*β* can upregulate the expression of adhesion molecules on endothelial cells, which bind to the leukocyte ligands and allow activated leukocyte entry into the CNS [16, 17]. Upon inflammation, endothelial cells also produce chemokines which recruit leukocytes into the CNS [18]. The traffic of immune cells through the BBB may initiate and/or contribute to a “vicious circle” in the disease process, resulting in progressive synaptic and neuronal dysfunction and neuronal loss in disorders such as Alzheimer's disease (AD), Parkinson's disease, amyotrophic lateral sclerosis (ALS), multiple sclerosis (MS), and neuroAIDS [19, 20].

### 2.2. Astrocytes

Astrocytes are a highly heterogeneous population of cells which regulate pH, the extracellular levels of neurotransmitters and ions, and energy metabolism. They are also involved in the formation and functioning of the blood-brain barrier (BBB) [21] and participate actively in neurotransmission [22]. In the small arteries, astrocytes contribute to neurovascular coupling, which synchronizes levels of neuronal metabolic demand with local cerebral blood flow regulation [23]. Abnormal astrocytic activity coupled with vascular instability has been observed in AD models [24].

In CNS insult situations, the typical response is a degree of reactive gliosis [25], an astrocytic response involving the positive gene regulation of cytoskeletal proteins such as glial fibrillary acidic protein (GFAP). The phenotypic changes include crumpled and bushy projections and large nuclei, culminating in hypertrophy of the cell body, hyperplasia, and functional alterations, in some cases resulting in glial scars [26]. Activated astrocytes also increase expression of the calcium-binding protein S100*β* [27, 28], an astrocyte activation marker particularly associated with the proliferation of astrocytes [29]. In streptozotocin- (STZ-) induced diabetes the phenotypic change in astrocytes and microglia glial cells is evidenced by increased expression of S100*β* and GFAP (astrocytic markers) and Iba-1 and MHC II receptors (reactive microglia markers) [30]. In addition, the death of neurons and such glial cells (the caspase-3 pathway) are also observed in the hippocampus, which is directly related to cognitive impairment [30, 31].

Reactive astrogliosis is a hallmark of PD and AD, supporting the hypothesis that astrocytes, together with microglial cells, play a central role in neurodegenerative diseases [32, 33]. Interestingly, attenuation of reactive gliosis by genetic ablation of the astrocyte intermediate filaments leads to more severe pathologies of AD, suggesting that reactive gliosis could have a protective role in the postacute and early chronic stage of neurodegenerative diseases (review in Pekny et al., 2014) [34].

Furthermore, astrocytes play an important role in central immunity. These cells either respond quickly to the presence of pathogens or other types of damage to the tissue (endogenous aggregated and oxidized proteins), assuming the role of immune cells by releasing cytokines and chemokines, influencing other cells, and modulating the BBB [26]. Astrocytes express toll-like receptors (TLRs) [35]. The in vitro activation of the astrocytic receptors TLRs 2, 4, 5, and 6 induces ROS production, IL-1*β*, IL-6, TNF-*α*, and glutamate, favoring neuronal damage [36]. Additionally, astrocytes from the brain and spinal cord of patients with MS display increased expression of TLR3 and TLR4 in inflammation regions [37]. Most TLRs initiate a signal via the myeloid differentiation primary response protein 88 (Myd88) and TRIFF, with consequent activation of MAP kinases and I*κ*B. The phosphorylation of I*κ*B results in translocation of NF-*κ*B to the nucleus, whereas the activation of MAP kinases results in phosphorylation of the AP-1 transcription factor. Activation of both signaling pathways culminates in the secretion of proinflammatory cytokine (IL-1*β*, IL-6, TNF-*α*, IL-12, and type I IFNs). Activated astrocytes can also produce chemokines (CCL and CXCL families) that recruit microglial cells, lymphocytes, and dendritic cells to the local of injuries (review in González et al., 2014) [38].

In the last decade, new evidence has been discovered about the importance of glial cells in acute and chronic inflammation of the brain. Hepatic encephalopathy (HE) is a major neurological complication of severe liver disease, characterized by impaired neurological function, including changes in personality, altered mood, diminished intellectual capacity, and abnormal muscle tone and tremor. The principal pathological change in chronic HE is characterized by Alzheimer type II astrocytosis [39]. Astrocytes are the main target of pathology in HE as they contain glutamine synthetase, the only enzyme in the brain that can remove ammonia [40]. Ammonia triggers inflammatory responses in microglia and the brain endothelial cells (ECs), most likely through the activation of the toll-like receptor-4 and consequent production of cytokines, and leads to severe astrocyte swelling/brain edema in acute hepatic encephalopathy [41]. Astrocyte swelling activates Erk-1/Erk-2- and p38-type MAP kinases, which may represent a signal for the proliferation and development of gliosis [42]. Proinflammatory cytokines (TNF-*α*, IL-1*β*, and IL-6), synthesized by astrocytes and microglia, induce disruption of the permeability barrier of the brain and can alter the normal balance and physiological function of cytokines in synaptic plasticity, learning, and memory functions [43–45]. Moreover, in a rat model of minimal hepatic encephalopathy (MHE), elevated levels of serum dopamine, released from cirrhotic livers, crossed the BBB and inactivated the Glutamate-NO-cGMP pathway in astrocytes, triggering memory impairment [46].

Evidence indicates that the cGMP-PKG pathway is involved in the regulation of astrocyte activity. NO/cGMP/PKG inhibits the expression of MMP-9 expression in LPS-stimulated rat primary astrocytes, suggesting that NO can downregulate MMP-9 in brain injury [47, 48]. According to these authors, MMP-9 expression is dependent on ERK 1/2 activation via NF-*κ*B. This data supports the hypothesis that the NO/cGMP/PKG pathway plays a role in astrocytic cells that contributes to the resolution of neuroinflammation.

### 2.3. Microglia

Microglia play a critical role in the innate and adaptive immune responses of the CNS. Microglia are derived from mesodermal/mesenchymal cells, which enter the brain parenchyma and acquire a specific ramified morphological phenotype described as “resting” microglia [49, 50]. Circulating monocytes provide another important source of microglia in the brain [51]. The physiological functions of microglia are important for maintaining homeostasis. They remodel synapses by phagocytosis [52], secret neurotrophic factors such as brain-derived neurotrophic factor (BNDF) [49], and remove accumulating debris and aggregated proteins [53]. However, when exposed to infections, injuries, or nervous system dysfunction, microglial cells undergo a complex activation process that converts them into activated microglial cells. The phenotypic changes after activation of the microglia are functionally identical to those observed in macrophages. In the absence of pathology, the “resting” microglia are cells with small bodies and long, thin processes (“ramified” phenotype). When activated, microglia lose the long extensions typical of inactive microglia and reveal stubby processes (“amoeboid” phenotype), finally exerting a phagocytic form [54, 55]. This physiological transformation is associated with changes in surface antigen expression and cytokine release, the suppressive effects of which may contribute to the impaired synaptic plasticity of neurodegenerative diseases [56].

Several neurodegenerative-derived molecules (aggregated amyloid-*β*, A*β*, *α*-synuclein of Lewy bodies in Parkinson's disease, mutant huntingtin, superoxide dismutase-1, and chromogranin A) can activate pattern recognition receptors (PRRs) expressed on the microglial surface, such as toll-like receptors (TLR2, TLR4, and TLR6), CD36, and triggering receptor expressed by myeloid cells 2 (TREM2). CD36, CD14, TLR2, TLR4, and TLR6 ligation activates the proinflammatory MyD88-NF-*κ*B signaling pathways, while TREM2 ligation increases the clearance of some aggregated molecules such as A*β*. Both pathways lead to formation of the NOD-, LRR-, and pyrin domain-containing 3 (NLRP3) inflammasome, which culminates with the secretion of active IL1-*β* or IL-18. NLRP3 inflammasome activation has been described in several neurodegenerative diseases such as AD and amyotrophic lateral sclerosis (for review, see Heneka et al., 2014) [56]. Activated microglial cells become a source of TNF-*α*, IL1-*β*, IL1-*α*, superoxide, nitric oxide (NO), chemokines, and glutamate, which can promote neuronal death. TNF-*α*, secreted by both microglia and astrocytes, can directly promote neuronal death by ligation to their correspondent receptors (TNFRs). Evidence indicates that TNF-*α* induces apoptosis in oligodendrocytes in demyelinating inflammatory diseases [57] and plays a fundamental role in neurodegeneration in PD and AD [38].

Another microglial neurotoxic mechanism is the excessive production of glutamate, which triggers synaptic and extrasynaptic NMDA receptors in neurons, causing neuronal death through increased calcium influx, a process denominated excitotoxicity involved in several neurodegenerative diseases [58]. As well as neuronal damage, glutamate also induces apoptosis of precursor, immature, and mature oligodendrocytes contributing to myelin damage in hypoxic-ischaemic pathologies [59]. Another mechanism of injury of high levels of glutamate in the brain is the release of TNF-*α* and IL1-*β* by microglia, which further aggravates the demyelinating process [60]. Microglial-derived interleukin 1 (IL-1) is also associated with phosphorylation and the aggregation of microtubule-associated protein tau (MAPT), a neuropathological feature of tau diseases [61].

Activated microglia contributes to neurodegenerative processes by secreting ROS and nitric oxide (NO) through NADPH [62], myeloperoxidase (MPO) [63], and inducible NO synthase (iNOS) activation [64]. Under conditions of oxidative stress reactive oxygen species (ROS) act as critical signal molecules that trigger an inflammatory response in the CNS through the activation of transcription factors, such NF-*κ*B and activator protein 1 (AP-1), promoting BBB damage and enabling immune cells to penetrate into the CNS [65, 66]. Furthermore, the upregulation of these enzymes increases the production of superoxide and NO, generating peroxynitrite, a potent oxidant that is thought to cause protein nitration. Increased levels of nitrated proteins represent a pathological event associated with several neurodegenerative diseases, such as amyotrophic lateral sclerosis, Parkinson's disease, and Alzheimer's disease (AD), demonstrating the potential involvement of reactive nitrogen species (RNS) in neurodegeneration [67, 68]. Another study showed that the ROS/ERK/JNK/MAPK signaling pathway contributes to microglial NO production [69]. The oxidative stress response of microglial cells is mostly due to the activity of NADPH oxidase, which appears to have a central role in the pathology of dopaminergic neuron death and the progression of PD [70]. Interestingly, Park et al. (2015) [71] proposed that although ROS involved in microglia activation are thought to be generated primarily by NADPH oxidase, mitochondria constitute a major source of ROS generation in LPS-mediated activated microglia cells.

A group of studies has shown evidence of bidirectional crosstalk between microglial cells and astrocytes involved in the physiopathology of neurodegenerative diseases. High levels of TNF-*α* produced by microglial cells can induce a dramatic glutamate release by astrocytes, leading to neuronal excitotoxicity [72]. Another important cellular interaction is microglia-neuron crosstalk. Intracellular chaperone heat shock protein 60 (HSP60) released from dying neurons activates microglial cells through the TLR4 and MyD88 pathway, leading to the synthesis of neurotoxic NO from microglia [73]. Similarly, the release of high mobility group box (HMGB1) by damaged neurons binds to the microglial Mac1 (macrophage antigen complex 1) and activates the nuclear factor-*κ*B pathway and NADPH oxidase, stimulating the production of multiple inflammatory and neurotoxic factors, in a vicious cycle that mediates chronic and progressive neurodegeneration [74].

### 2.4. Oligodendrocytes

Oligodendrocytes are specialized cells responsible for myelin synthesis and maintenance and provide a supporting role for neurons [75, 76]. There is intense crosstalk between oligodendrocytes and the other glial cells. Activated microglial cells are considered key initiators of the demyelination process mediated by ROS, which is the most important damage factor of oligodendrocytes. Dysfunction of oligodendrocytes and myelin abnormalities are found in a wide variety of neurological disorders and may be involved in the pathophysiology of several diseases, including genetic leukodystrophies [77], schizophrenia and bipolar disorder [78, 79], brain injury [80], and endocrine and metabolic abnormalities [81, 82] and in neurodegenerative conditions such as strokes [83, 84], Parkinson's disease [85], Alzheimer's disease [86–88], and multiple sclerosis [89] and in diabetic encephalopathy [90].

Oligodendrocytes are very vulnerable to ROS and RNS because of their low concentration or the activity of antioxidant enzymes (superoxide dismutase, glutathione reductase/peroxidase) [66], resulting in neuronal death [91]. During pathological processes, the insult to white matter, originating from oxidative stress, inflammation, and mechanical injury, leads to degenerative loss (demyelination) or inadequate or abnormal formation of myelin (hypomyelination) [92]. The major consequences include oligodendrocyte death and the destruction of the myelin sheath, recruitment failure, and/or the differentiation of oligodendrocyte precursor cells (OPCs) during remyelination [93–96].

Oxidative stress is widely accepted as playing a key mediatory role in the development and progression of diabetes and its complications [97]. Diabetic encephalopathy can induce CNS disorders such as impairment of learning and memory [30, 98–100], dementia [101], apoptosis of hippocampal neurons [31, 102], increased permeability of the blood-brain barrier [103], edema, disarrangement of the myelin sheath, and oligodendrocyte abnormalities in the cerebral cortex, cerebellum, and hippocampus [102].

Oxidative stress also exerts a critical role in AD pathogenesis [104–106]. A*β* induces oligodendrocyte dysfunction and apoptosis through oxidative stress [107, 108]. During the early stages of AD, toxicity mediated beta-amyloid induces the destruction of myelin in cognition related areas in experimental models [87, 109]. Similarly, damage to the myelin associated with aging is clinically associated with neurological deficits, such as a delay in cognitive processing and memory [110]. Initially, deposits of A*β* plaques appear to enhance the survival, proliferation, and differentiation of OPCs [111]. However, other studies have reported the involvement of A*β* in acute inflammation, oxidative stress, and neuronal apoptosis [65, 112–114].

Oligodendrocyte injury is also evident in other demyelinating diseases. In MS, Th1 and Th17 have been shown to be the main pathogenic T cells, as they promote BBB disruption, demyelination, and neurodegeneration [104, 115]. The process is the initial response of the endothelium of the blood-brain barrier, as it is capable of expressing MHC class II antigens, allowing the migration of CD4+ and CD8+ T cells to the perivascular space [116, 117]. As a result, the activated macrophages and microglia release large amounts of inflammatory cytokines such as IL-1*β* and TNF-*α*, thereby amplifying the proinflammatory autoimmune reaction [74]. Both cytokines are involved in oligodendrocyte damage via the MAPK signaling pathway. The binding of TNF-*α* to its receptor TNF-R1 induces oligodendrocyte apoptosis, whereas the binding of IL-1*β* to IL-1R1 delays remyelination [118]. In an attempt to repair myelin damage, oligodendrocyte precursor cells (OPCs) transform into mature oligodendrocytes, promoting remyelination. In more advanced stages of the disease, however, OPCs also undergo apoptosis [119].

Oligodendrocytes are not immunological inert cells but secrete a wide range of inflammatory mediators, expressing receptors to such factors. They produce the proinflammatory cytokines IL1-*β* and IL-6 and the chemokines CCL2 and IL-8, which are involved in the recruitment of immune cells during acute inflammation [120, 121]. In MS experimental models, dying oligodendrocytes also express increased COX-2 levels at the onset of demyelination, which seems to render these cells more susceptible to death by glutamate-mediated excitotoxicity [122].

In summary, glial cells may serve as a potential therapeutic target for neuroinflammatory and neurodegenerative disorders and disturbances in learning processes and memory.

## 3. Cyclic Nucleotide Signaling and Neuroinflammation/Neurodegeneration

Cyclic nucleotides, cyclic adenosine monophosphate (cAMP), and cyclic guanosine monophosphate (cGMP) exert many physiological roles such as the regulation of ion channels, relaxation of smooth muscle, immunomodulation, platelet aggregation, phototransduction, neuronal survival, and consolidation of memory [123–125].

cAMP is synthesized from adenosine 5′-triphosphate (ATP) by membrane-bound adenylyl cyclase (AC), which is mainly regulated in neurons by G-proteins and additionally stimulated by and calmodulin. The main transducer of cAMP signals is the cAMP-dependent protein kinase A (PKA) [126].

Synthesis of cGMP is regulated by membrane-bound/particulate (pGC) and cytosolic/soluble (sGC) guanylate cyclases, which convert guanosine 5′-triphosphate (GTP) into cGMP. The physiological effects of cGMP activities are determined by three types of intracellular receptors: cGMP dependent kinases (PKG), ion channels regulated by cGMP, and PDEs regulated by cGMP. cGMP can also activate the cAMP pathway by activating the cAMP intracellular receptors, such as its main target protein kinase, PKA [127].

Both cAMP and cGMP can alter cell function by activating or inactivating proteins by phosphorylation. The most important regulation of cyclic nucleotides is achieved by the breakdown of cAMP and cGMP in their inactive forms, 5′AMP and 5′GMP, respectively, by phosphodiesterases (PDEs) [4]. In several cell types, cGMP modulates the concentration of cAMP by activating or inhibiting cAMP-specific phosphodiesterases (PDEs) [127].

In the central nervous system (CNS), cGMP plays an important role as a mediator of the action of nitric oxide (NO) and natriuretic peptides. NO is a gaseous free radical that acts as an important sign of intra- and extracellular processes [128] and is synthesized intracellularly by three isoforms of the nitric oxide synthase enzyme (NOS): (1) dependent Ca^+2^ constitutive forms, consisting of the endothelial form or type III (eNOS or NOS-III) and the neuronal form or type I (n-NOS or NOS-I), present in endothelial cells, neurons, and glial cells, which produce NO under physiological conditions; (2) Ca^+2^ independent inducible form (i-NOS) or type II, present in macrophages, hepatocytes, smooth muscle, endothelium, and glial cells, which produce NO after immunological stimulation (i.e., IFN-*γ*, TNF-*α*, and LPS) [5].

The role of NO in inflammation is complex. At micromolar range, NO produced by iNOS exerts cytotoxic and proinflammatory effects that are opposite to those induced by low nanomolar concentrations of NO produced by the eNOS isoform, which exhibits anti-inflammatory effects via the cGMP-PKG pathway [129].

Some studies indicate that NO derived from both nNOS and eNOS, but not the iNOS isoform, is critical in the regulation of leukocyte-endothelial cell interactions [129–131]. Nitric oxide (NO) modulates leukocyte adherence and recruitment on the vascular endothelium, exerting a cytoprotective and antithrombotic role. The anti-inflammatory effects of NO are mediated predominantly via the activation of sGC/cGMP. The production of cGMP causes specific downregulation of the expression of P-selectin on endothelial cells and platelets to prevent leukocyte rolling [132].

Moreover, it has been reported that the intracellular accumulation of cGMP in different models of inflammation reduces the production of proinflammatory cytokines such as IFN-*γ*, TNF-*α*, and interleukins (ILs), lowers oxidative stress, and diminishes the production of chemokines and chemokine receptors such as monocyte chemoattractant protein (MCP-1) and its receptor CCR2. Therefore, intracellular levels of cGMP exert a role in modulating inflammatory response [133, 134].

Inflammatory response is a tightly regulated physiological process, involving the orchestrated expression of inflammatory mediators. cAMP interferes with the function of the proinflammatory transcription factor Nuclear Factor-kappaB (NF-*κ*B), as a the result of the inhibition of I*κ*B degradation due to blocking of IKK activity by cAMP/PKA [135, 136] or enhanced levels of resynthesized I*κ*B [137]. NF-*κ*B plays a crucial role in switching on the gene expression of a plethora of inflammatory and immune mediators. Cyclic AMP modulates NF-*κ*B when activated by typical stimuli, such as proinflammatory cytokines, B- and T-cell activators, pathogen-associated molecular patterns (PAMPs), and oxidative stress, but also has an effect on the triggering of NF-*κ*B by less common activators such as amyloidogenic peptides, thrombin, and high levels of glucose. However, other observations suggest that cAMP/PKA can induce NF-*κ*B transactivation. A possible explanation for the seemingly conflicting effects of cAMP/PKA on NF-*κ*B activation may lie in the existence of different PKA pools, with distinct subcellular localization and different functions (review in Gerlo et al., 2011) [126].

The NO-cGMP pathway can directly inhibit vascular NF-*κ*B inflammatory activity by increasing the cytoplasmic and nuclear levels of I*κ*B*α* expression [138] and inhibition of NF-*κ*B binding [139] or indirectly by activating the kinase-A protein in the cGMP-dependent pathway [133]. Moreover, eNOS regulates NF-*κ*B expression in a negative feedback mechanism, limiting local inflammation [140].

The NO/cGMP/PKG pathway appears to play an essential role in preventing the activation of a proapoptotic pathway, thus promoting neural cell survival. This neuroprotective mechanism may be especially important during brain ischemia, inflammation, or trauma [141]. In retinal neuroglial progenitor cells, NO/cGMP/PKG antiapoptotic cascade is activated through the cAMP-responsive element binding protein (CREB) [142], the transcription factor involved with neurotransmitters, growth factors, and other signaling molecules with essential functions for long-lasting changes in synaptic plasticity, which mediates the conversion of short-term memory to long-term memory and neuronal survival [143, 144].

The brain-derived neurotrophic factor (BDNF) is one of the major gene products of CREB-mediated transcription that is upregulated on cyclic nucleotide level elevation. The neurotrophin BDNF and its major receptor Tr*κ*B have the most abundant and widespread expression in the developing and adult mammalian brain and have a critical role in the differentiation and survival of neurons of the CNS and in long-term potentiation (LTP), a form of synaptic plasticity. The activation of the BDNF/ Tr*κ*B pathway is directly implicated in the rise of intracellular Ca^+2^ via its release from intracellular stores, and in the activation of the Ca^+2^-calmodulin dependent kinase, CaMKII. The elevation of intracellular Ca^+2^ is one of the most important biochemical outcomes of BDNF signaling in the postsynaptic cell (review in Cunha et al., 2010) [145]. Moreover, BDNF/ Tr*κ*B signaling activates phosphatidylinositol-3-kinase/Akt cascades, responsible for neuronal survival via bcl-2 activation and Bad inactivation [146]. The inhibition of CREB phosphorylation may impair synaptic plasticity and apoptosis due a reduction of BDNF levels.

## 4. Effect of Phosphodiesterase-5 Inhibitors on Neuroinflammation and Neurodegeneration

### 4.1. Neurogenesis and Antioxidant Activity

Neurogenesis is the biological process of generating new neurons from progenitor or neural stem cells (NSCs). NSCs proliferate in two main regions of the adult mammalian brain: the subventricular zone (SVZ) of the lateral ventricles and the subgranular zone (SGZ) of the dentate gyrus of the hippocampus [147]. Neurogenesis can be influenced by several factors, including the release of growth factors, serotonin, estrogen, and glucocorticoids, among others [148, 149]. PDE-5 inhibitors have been reported to promote neurogenesis [113, 150].

Santos et al. (2014) [147] reported that inhibitors with different selectivity for PDE5, such as T0156, sildenafil, and zaprinast, enhanced the proliferation of neural stem cells (NSCs), the first step of neurogenesis, through the sGC/PKG/ERK/MAPK pathway, with the exception of sildenafil, which did not alter ERK1/2 phosphorylation. A similar lack of an effect of ERK1/2 on phosphorylation by sildenafil was previously described by Zhang et al. (2005) [151], who suggested that the increase in cGMP levels via the inhibition of PDE5 activity enhances neurogenesis through the PI3K/Akt pathway. As already mentioned, the NO/cGMP/PKG/CREB/BDNF pathway has a fundamental role in neurogenesis and synaptic plasticity by activating PI3K/Akt.

Several studies have reported that PDE5Is inhibitors have a neuroprotective effect though elevating cGMP brain levels. Sildenafil has been found to reduce the neurologic deficit, improve neurogenesis and memory, and promote functional recovery after a stroke and focal cerebral ischemia in young and aged rats [152, 153]. Tadalafil also improved neurogenesis in an embolic stroke model in rats [153]. Another study demonstrated that pretreatment with tadalafil attenuated the deleterious effect of cerebral ischemia-reperfusion on infarct size, nitrosative and oxidative stress, memory, and motor coordination. These effects were attenuated by administration of L-NAME, a nonselective nitric oxide synthase inhibitor [154].

Antioxidant activity has also been described with the use of vardenafil on cerebral vasospasm in an experimental rat subarachnoid hemorrhage model, which induced dose dependent vasodilation of the basilar artery and also had an antioxidant effect by reducing lipid peroxidation [155].

Sildenafil also has an antioxidant and anti-inflammatory effect. An in vitro study using N9 microglial cells demonstrated that sildenafil suppressed NO, interleukin 1*β* (IL-1*β*), and tumor necrosis factor *α* (TNF-*α*) production induced by LPS. Sildenafil also blocked I*κ*B*α* phosphorylation and degradation, inhibited the phosphorylation of mitogen-activated protein kinases (MAPKs), extracellular signal-regulated kinases 1 and 2 (ERK1/2), p38 MAPK, and c- Jun N-terminal kinase (JNK). Moreover, sildenafil downregulated gp91phox, a critical and catalytic subunit of NADPH oxidase, and levels of intracellular ROS. A possible mechanism for the anti-inflammatory and antioxidant effects of sildenafil may be at least in part due to suppression of the MAPKs/NF-*κ*B pathways through the inhibition of NADPH oxidase-mediated ROS generation [156].

Chronic administration of sildenafil in diabetic type II (T2DM) patients reduces levels of endothelin, C-reactive protein, interleukin-6, intercellular adhesion molecules (ICAM), and vascular adhesion molecules (VCAM), as well as reducing nitrate/nitrite levels [157]. Recent clinical research showed that treatment of T2DM patients with sildenafil for three months reduced the endothelial function marker P-selectin and exerted a beneficial effect on glycometabolic control [158].

Similarly, daily administration of tadalafil also reduced circulating levels of the proinflammatory cytokines TNF-*α* and IL-1*β*, improved fasting glucose levels, and reduced infarct size following I/R injury in the heart of diabetic type II mice [159]. The diabetic myocardium is exposed to intense oxidative stress that can eventually lead to cardiac tissue injury and dysfunction [160]. Chronic treatment with tadalafil reduced ROS production, cardiac NADPH oxidase activity, lipid peroxidation, and oxidized glutathione [161]. Clinical studies also confirmed that the beneficial effects of tadalafil treatment on vascular function occur via improvement of endothelial function markers such as C-reactive protein, endothelin-1, and ICAM-1 [162].

### 4.2. Neuropathy and Motor Neuron Diseases

The early impairment of endothelial NO in diabetic patients may contribute to increased susceptibility to damage to neurons that are normally protected by the NO/cGMP/PKG signaling pathway, which may be responsible, in part, for diabetic polyneuropathies (review in Fiscus, 2002) [141]. Diabetic peripheral neuropathy is characterized by the loss and/or degeneration of neurons, Schwann cells, and neuronal fibers and by the slowing of nerve conduction velocities [163]. Diabetic patients treated with sildenafil reported an improvement in peripheral neuropathy symptoms [164]. In rodents with diabetic peripheral neuropathy, treatment with sildenafil improved blood supply to the vasa nervorum and functional outcome through the nitric oxide- (NO-) cGMP pathway [165].

According to the elegant studies performed by Wang et al. (2011) [166], diabetic type II mice present upregulated PDE5 expression in the sciatic nerve, whereas myelin sheath thickness, myelin basic protein (MBP), and the subcutaneous nerve fibers are significantly reduced. Treatment with sildenafil significantly counteracted these effects and concomitantly improved neurological function, assayed by motor and sensory conducting velocities and thermal and mechanical noxious stimuli. In vivo and in vitro analysis demonstrated that PDE5/cGMP regulates BDNF expression in the Schwann cells of diabetic mice, enhancing myelin formation in the sciatic nerve. The same research group also studied the therapeutic effect of sildenafil in middle aged diabetic mice with long-term peripheral neuropathy, concluding that sildenafil is likely to contribute to the amelioration of nerve function through angiopoietin-1 (Ang1) and its receptor Tie-2 signaling, promoting the beneficial effects of sildenafil on neurovascular function in diabetic mice [69].

It is apparent that increases in cGMP levels favor the proliferation of motor neurons. Amyotrophic lateral sclerosis (ALS), a neurodegenerative disorder characterized by the rapid degeneration of motor neurons, has also been the subject of many studies. While the pathogenesis of ALS is clear, the results of such studies suggest the involvement of excitotoxicity [167], peroxynitrite toxicity [168], or other oxidative damage [169]. An in vitro study using motor neuron culture showed that both PDE-5 inhibitors such as sildenafil and 8q-cGMP, an analogue of cGMP, provided neuroprotection against neurotoxicity induced by reactive oxygen species (ROS) and could therefore be a possible therapeutic tool for the treatment of ALS [170].

Another disease that causes impairment of movement is Huntington's disease (HD), an autosomal dominant neurodegenerative disorder caused by an expanded CAG repeat in the coding region of the huntingtin gene. Drugs such as (PDE) inhibitors targeted at counteracting loss of CREB function and decreased BDNF have been considered as powerful tools for the treatment of HD [171]. Some studies showed that rolipram PDE-4 inhibitors are able to exert a neuroprotective effect and to significantly increase levels of activated CREB in the striatal spiny neurons, in a surgical model of HD [172, 173]. There are also reports that treatment of HD with PDE-10 inhibitors reduces the death of cortical neurons and increased phosphorylation of CREB and BDNF levels (review in Fusco and Giampà, 2015) [174]. Similarly, Puerta et al. (2010) [175], showed that sildenafil and vardenafil can improve neurological symptoms, reduce neuronal death, and increase levels of phosphorylated CREB in a HD model, indicating a possible neuroprotective effect.

### 4.3. Demyelinating Diseases

In demyelinating diseases, important functions such as electrical conduction, connectivity, and axolemmal organization are compromised. Consequently, the injured axons are unable to function efficiently, leading to severe psychomotor deficits [176]. The demyelination process is usually accompanied by an inflammatory condition caused by the release of cytokines and activation of glia cells (astrocytes and microglia), leading to the death of oligodendrocytes (review in Peferoen et al., 2014) [118].

Multiple sclerosis (MS) is a chronic immune-inflammatory disease of the central nervous system (CNS) characterized by demyelination of white matter and axonal injury. The action of sildenafil in improving the clinical symptoms of multiple sclerosis (MS) patients initially was assigned to neurogenesis induction, but recent information also points to the role of the drug as a modulator of inflammation and protection of the myelin sheath [6, 177–179].

Sildenafil improved clinical signs and neuropathology in a murine model of multiple sclerosis (EAE), promoting remyelination and reducing infiltration of CD3+ leukocytes and microglia/macrophages activation [177]. Recently, Pifarré et al. (2014) [180] showed that daily treatment with sildenafil from the onset of symptoms of EAE prevented further clinical deterioration by stimulating immunomodulatory and neuroprotective mechanisms. According to these authors, early administration of sildenafil downregulated adaptative immune responses switching from the M1 to the M2 phenotype in microglia/macrophages. Furthermore, in vitro analyses of splenocytes found that sildenafil downregulated Th1/Th2/Th17 responses, while upregulating Tregs.

Additionally, Nunes et al. (2012) [178] demonstrated that sildenafil inhibited the demyelination process and reduced micro- and astrogliosis and expression of proinflammatory cytokines (TNF-*α*, IFN-*γ*, and IL-2 IL1*β*) in a cuprizone-induced demyelination model. In another study by the same group, sildenafil increased levels of protein expressed by oligodendrocytes and MBP (myelin basic protein) and restored the morphology of the myelin sheath, indicating remyelination. In addition, sildenafil induced OPC differentiation into mature oligodendrocytes, demonstrated by increased GST-pi (marker of mature oligodendrocytes) [6]. Sildenafil also induced myelin repair and myelin debris clearance, possibly associated with the release by the microglia of MCP-1 chemokine and metalloproteinase MMP-9 (unpublished data).

## 5. Cyclic Nucleotides Signaling Pathways and Cognition

Memory consolidation is the process by which newly acquired information is stabilized and stored [181]. Consolidation is a very complex brain function which requires specific molecular mechanisms and evolves two stages over time: a short-term and a long-term phase, which differ in their dependence on new protein synthesis [182]. Long-term potentiation (LTP), an electrophysiological measure involving the sustained increase in synaptic efficacy of the hippocampal synapses, is the neurophysiological correlate of memory [183]. LTP and memory share the same molecular mechanism, and, therefore, changes in synaptic strength can also be divided into two temporally and mechanistically distinct phases. A single train of high frequency stimulation (e.g., 100 Hz, 1 s), which mimics the physiological bursts of neuronal activity in the hippocampus, induces a transient increase in synaptic efficacy called early phase LTP (E-LTP). This early phase involves the short-term modification of preexisting synapses and posttranscriptional modification events [183, 184]. In contrast, repeated high-frequency stimulation (e.g., four trains of 100 Hz every 10 min) induces long-lasting, late-phase LTP (L-LTP) [185]. L-LTP, as well as long-term memory, induces the synthesis of new proteins which are responsible for the stable structural changes required for memory trace stabilization [186].

Both the cAMP/PKA and cGMP/PKG signal transduction pathways regulate the molecular mechanism that underlies LTP. It had been suggested that the two pathways were differentially involved in the distinct phases of the memory consolidation process. Whereas cAMP was described as being involved in the formation of late-LTP (long-lasting synaptic changes) [187], cGMP was related to the transient early-LTP phase (labile synaptic changes) [188, 189]. Bacskai et al. (1993) [190] demonstrated how cAMP and PKA participate in the RNA and long-term protein synthesis-dependent process. An increase in cAMP concentration can induce gene transcription through the phosphorylation of CREB (Ser133) [191]. Interestingly, the activation of CREB can also be triggered by cGMP, by increasing intracellular Ca^+2^ levels [192]. The cGMP and PKG pathways cause the release of Ca^+2^ from ryanodine-sensitive stores, and when the Ca^+2^ signal is sufficiently large, it causes phosphorylation of CREB and can induce LTP in parallel with PKA [193]. cGMP-induced potentiation was blocked by protein inhibitors, RNA synthesis, and a PKG inhibitor, but not by a PKA inhibitor, suggesting that it is PKA-independent [192]. Nonetheless, it has been shown that these merge at some stage as both PKG- and PKA-induced late-phase potentiation are blocked by protein inhibitors and RNA synthesis [185].

In the same way, whereas cGMP is implicated in early memory consolidation processes, a role has been attributed to cAMP in late memory consolidation [194, 195]. However, behavioral studies have shown that NO was also involved in long-term memory [196], raising the question of whether cGMP signaling might also be involved. In fact it has recently been demonstrated that NO contributes to long-term memory via the activation of soluble guanylyl cyclase (sGC), cGMP-dependent protein kinase, and CRE-binding protein (CREB) phosphorylation [197]. In a recent study, Bollen and Prickaerts, 2012 [4], showed that cAMP and cGMP signaling act independently to improve memory formation and that cGMP/PKG signaling mediates both early and late memory consolidation, whereas cAMP/PKA signaling mediates late consolidation. Importantly, the cGMP/PKG pathway requires cAMP signaling to enhance consolidation, implying that this is the common pathway in long-term memory formation.

Thus, the late protein synthesis-dependent phase of LTP involves the induction of immediate early genes via CREB phosphorylation, which is mediated in part via PKA [198, 199] and also PKG [200, 201]. New synthesized proteins are required for the growth of new synapses, which may underlie the nootropic effects of PDE inhibitors.

Other downstream targets of PKG have been described in addition to the canonical cGMP/PKG/CREB signaling pathway. While most previous studies were carried out in the hippocampus, in the amygdala the NO-cGMP-PKG signaling pathway regulates LTP and promotes fear memory consolidation via activation of ERK/MAPK signaling, which promotes ERK-driven immediate early gene expression [202, 203]. The cGMP pathway also plays an important role in the specific signaling mechanism that promotes branch formation in neurons [204]. In this case, a direct link has been found between cGMP signaling and GSK-3*β*, a kinase known to phosphorylate cytoskeletal proteins in neurons [205]. GSK-3*β* is an important kinase involved in tau pathology associated with AD and therapeutic approaches aimed at the inhibition of these kinases present a novel perspective for the management of AD [206]. It has been hypothesized that the inactivation of GSK3*β* and the consequent decrease in tau phosphorylation also contribute to the restoration of cognitive function caused by PDE5 inhibitors in AD mice (review in García-Osta et al., 2012) [207].

## 6. Phosphodiesterase Inhibitors 5 on Cognition

PDEs inhibitors can play a major role in memory function, regulating cell signaling by increasing the concentration of cGMP or cAMP throughout the brain [207]. One of the first studies investigating the effects of PDE inhibitors as memory enhancers in AD patients demonstrated that vinpocetine, a PDE1 inhibitor, failed to improve cognition or slow the rate of memory decline [208]. However, more recent studies have demonstrated the effectiveness of PDE3 (cilostazol), PDE4 (rolipram), or PDE5 (sildenafil or tadalafil) inhibitors in reversing memory impairments in several mouse models of AD [10, 209–212]. The prominent expression of PDE5 in the smooth muscle of the meningeal arteries and in blood vessels suggests that a peripheral effect of PDE5 inhibitors, which may lead to an improvement in cerebral blood flow, may also contribute to the procognitive action of these drugs [211]. However, some authors demonstrated that systemic administration of vardenafil does not affect cerebral blood flow in the hippocampus and even decreases it in some of the brain areas studied [213]. Moreover, a recent report by Akkerman et al., 2015, demonstrated the effectiveness of vardenafil after intracerebrovascular administration, providing evidence that PDE5 inhibitors enhance memory via a central mechanism [9]. Nevertheless, the underlying mechanism has yet to be fully elucidated.

In physiological conditions, PDE5 inhibitors have a more pronounced effect as memory enhancers in aged mice than in young mice. Recently, a report has demonstrated that chronic treatment with sildenafil improved memory in the object recognition and Morris Water Maze (MWM) task in aged mice but not in young mice [214]. The effect on memory is paralleled with an increase in synaptic plasticity and a restoration of pCREB levels in the hippocampus [214]. Interestingly, in the senescence-accelerated prone mouse (SAMP8), a model of age-related cognitive decline, chronic treatment with sildenafil attenuated learning and spatial memory impairments in the MWM [215]. The authors argued that this nootropic effect in SAMP8 mice is mediated by a decrease in tau hyperphosphorylation, an effect that could be mediated by the modulation of the Cdk5/p25 and Akt/GSK-3*β* pathways by sildenafil [215]. Similar results were obtained with chronic tadalafil treatment in aged mice, where tadalafil enhanced memory by increasing BDNF levels and dendritic spine density in apical dendrites on CA1 hippocampal pyramidal neurons, which may contribute to enhancing learning and memory process [11].

In relation to human reports, udenafil has been used to study the effect of repeated dosing of PDE5-I on cognition [216, 217]. In a pilot study involving 60 men with erectile dysfunction, udenafil improved cognitive function and depression after two months of daily dosing treatment. The authors argued that the effect was mediated by an increase in the NO-cGMP signaling pathway, increased glucose and oxygen delivery to the brain, and increase in self-esteem in patients [216].

Aging and accumulation of amyloid *β* (A*β*) peptides are important risk factors for the development of dementia. It has been reported that a decrease in the basal level of cGMP occurs in the brain with aging [218]. cGMP synthesis in the central nervous system occurs mainly through the activation of NO-dependent soluble guanylate cyclase (sGC). At the same time, NO formation is typically coupled to the activation of NMDA receptors meaning, therefore, that the activation of the NMDA receptor significantly stimulates NO/cGMP production in the hippocampus. On the other hand, cGMP hydrolysis is regulated by PDEs, specifically PDE2, PDE5, and PDE9, which are the most prevalently expressed in the brain. Thus, a decrease in cGMP could be a consequence of a more active degradation of cGMP by PDEs and/or a decrease in NMDA receptor-mediated cGMP formation in the aged brain when compared with the adult brain. The accumulation of A*β* peptides in AD can also decrease the NO/cGMP dependent signal transduction mediated by NMDA receptors [219]. Furthermore, changes in the expression and activity of both constitutive NOS isoforms, eNOS and nNOS, have been implicated in the decline of cognitive functions in the senescent brain by decreasing cGMP synthesis [219, 220].

Due to the possible regulation of the cGMP/PKG/CREB and cGMP/PKG/pGSK3 *β* pathways through the increase in levels of cGMP in the brain, PDE5 inhibitors appear to be good candidates for AD treatment. The expression of PDE5 in brain areas involved in cognition (e.g., the hippocampus and cortex) supports this hypothesis [11]. Nevertheless, the lack of availability of good brain-penetrant PDE5 inhibitors has been crucial to the carrying out of studies in AD animal models. At the same time, although currently available selective PDE5 inhibitors have low blood-brain barrier (BBB) permeability, tadalafil and sildenafil have been used in chronic treatments and in a range of AD models, confirming their efficacy in reversing cognitive impairment [10, 207, 211]. Increased cGMP levels have been detected in the brain after the administration of tadalafil and sildenafil, confirming that both drugs cross the BBB and reach the brain at a sufficient concentration to inhibit PDE5 [3, 11], the stimulation of the cGMP/PKG pathway and the restoration of CREB signaling may underlie the therapeutic effect of PDE5 inhibitors in AD. The activation of CREB promotes the transcription of genes, such as BDNF, which, as has been proposed, contributes to the neuroprotective effects of sildenafil [175]. Indeed, the upregulation of BDNF has also been observed in the hippocampus after chronic sildenafil treatment in a mouse model of AD. The full mechanism of action, however, has yet to be elucidated [211].

The downstream activation of CREB by the NO/cGMP pathway provides an interesting link to cognitive dysfunction and decreased synaptic plasticity in AD. The NO-cGMP-PKG signaling pathway contributes to CREB phosphorylation and LTP in the hippocampus, evidently acting in parallel with PKA and MAP kinase [192]. In fact, dysfunction in CREB signaling contributes to the pathology of AD, leading to synaptic dysfunction and cognitive impairment in both AD patients and AD animal models [209, 221]. It has been suggested that in AD, A*β* impaired synaptic plasticity by downregulating the NO/cGMP/PKG/CREB pathway in hippocampal slices [222]. It has been found that A*β* decreases the activity of sGC, and therefore the synthesis of cGMP in brain astroglial cells and in the temporal cortex of AD patients [223, 224]. It has recently been found that cGMP levels are significantly lower in the Cerebral Spinal Fluid (CSF) of AD patients compared with nondemented controls. Importantly, a significant association was found between cGMP levels and cognitive decline and A*β*42 levels in AD patients. The authors also identified an association between a decrease in cGMP and an increase in PDE5 expression in the temporal cortex of AD patients [225]. Taken together these findings suggest that the cGMP/PKG/CREB pathway is downregulated in AD and therapeutic strategies aimed at increasing cGMP seem to be a good option in dementia associated with aging and AD. Moreover, downregulation of the AC/cAMP/PKA pathway can also account for a loss of synaptic plasticity and memory in AD patients [226].

The beneficial effect of PDE-5 inhibitors in reducing A*β* levels is controversial. While some studies have demonstrated the beneficial effects of PDE5 inhibitors in reducing A*β* levels in different mouse models [10, 218, 227, 228], other authors have argued that PDE5 inhibitors did not affect the A*β* burden [11, 211]. This discrepancy may be due to differences in animal models and the severity of the amyloid pathology. Some authors argued that a decrease in amyloidogenic APP processing, and consequent A*β* formation, is induced by the inhibition of PDE5 [227, 229]. Nevertheless, the mechanisms by which PDE5 inhibitors decrease A*β* levels are not yet clear. In terms of tau pathology, another major pathological hallmark of AD, most authors agree that PDE5 inhibitors reduce levels of phosphorylated tau through the inhibition of GSK3beta [11, 211, 215].

In addition to sildenafil, tadalafil, and vardenafil, the most commonly used PDE5 inhibitors, other novel selective PDE5-Is have recently been identified. A selective quinoline-based compound with an IC50 of 0.27 nM and favorable permeability has also been shown to recover memory defects in a mouse model of AD [230]. Icariin, a flavonoid extracted from a Chinese herb (Berberidaceae* Epimedium* L.), has been described as an effective PDE5 inhibitor that is also an effective AD reversion phenotype in mice, through the stimulation of the NO/cGMP signaling pathway in the brain [218]. Finally, Yonkenafil, a novel PDE5 inhibitor [231] which has a strong inhibiting effect against PDE-5 (IC50 of 2.01 nM), improved cognitive function and ameliorated the amyloid burden in an APP/PS1 transgenic mice model, by inhibiting the activation of glial cells and restoring neurogenesis [231].

## 7. Concluding Remarks

In recent years, knowledge about NO-cGMP signaling has emerged as a promising target for neuroinflammation and cognitive disorders. Special attention, however, must be paid to the effect of NO-cGMP on glial cells, particularly oligodendrocytes, as little is known about their immunological function or their possible crosstalk with microglia and astrocytes in the progression of neuroinflammatory and demyelinating diseases. Moreover, little is known about the relationship between NO-cGMP and glial cells in the development of some important neurodiseases such as PD, HD, and ALS. Given the crucial role of NO-cGMP in neuroinflammation and cognition, there is a wide range of possible therapeutic applications of PDE5-Is. Furthermore, as the majority of studies to date have concentrated on sildenafil, the potential of other inhibitors, such as tadalafil, which has a long-lasting half-life and crosses the BBB, remains underexplored. Increased knowledge of this subject will provide a conceptual framework for the use of PDE5 inhibitors and the design and delivery of novel selective agents.
